# Case report: Remission of chronic low back pain and oral dysesthesia comorbid with attention deficit/hyperactivity disorder by treatment with atomoxetine and pramipexole

**DOI:** 10.3389/fpain.2023.1159134

**Published:** 2023-06-05

**Authors:** Satoshi Kasahara, Yuichi Kato, Miwako Takahashi, Ko Matsudaira, Naoko Sato, Shin-Ichi Niwa, Toshimitsu Momose, Kanji Uchida

**Affiliations:** ^1^ Department of Anesthesiology and Pain Relief Center, The University of Tokyo Hospital, Tokyo, Japan; ^2^ Department of Pain Medicine, Fukushima Medical University School of Medicine, Fukushima, Japan; ^3^ Department of Pediatric Dentistry, School of Life Dentistry at Tokyo, Nippon Dental University, Tokyo, Japan; ^4^ Institute for Quantum Medical Science, National Institutes for Quantum Science and Technology, Chiba, Japan; ^5^ Nursing Department, The University of Tokyo Hospital, Tokyo, Japan; ^6^ Department of Psychiatry, Aizu Medical Center, Fukushima Medical University, Fukushima, Japan; ^7^ Institute of Engineering Innovation, School of Engineering, The University of Tokyo, Tokyo, Japan

**Keywords:** oral dysesthesia, low back pain, chronic overlapping pain conditions, adult ADHD, atomoxetine, pramipexole, SPECT, Conners' adult ADHD rating scale

## Abstract

**Introduction:**

Oral dysesthesia is a disease characterized by pain and/or abnormal sensations in the oral region, without any organic abnormality. Its symptoms include pain, and it is considered to be a disorder associated with idiopathic oral-facial pain. It is also known that idiopathic oral-facial pain tends to coexist with chronic musculoskeletal pain, including low back pain, even before its onset. Such coexisting idiopathic pain conditions are also called chronic overlapping pain conditions (COPCs). In general, COPCs are often refractory to treatment. Recently, it has been reported that attention deficit hyperactivity disorder (ADHD) is associated with many COPCs, such as pain in the facial and lower back regions and so on. However, there are no reports of (1) ADHD as a comorbidity with oral dysesthesia (OD) or (2) of the therapeutic effects of ADHD medications or dopamine agonists on low back pain and OD or an (3) evaluation of cerebral blood flow over time after treatment with these medications for OD and low back pain.

**Case Presentation:**

In this study, we report the case of an 80-year-old man with OD and chronic low back pain that persisted for more than 25 years. His OD and chronic back pain were refractory to standard treatment, prevented him from continuing work, and tended to be exacerbated by conflicts in his relationship with his son. In recent years, ADHD has often been found to be comorbid with chronic pain, and ADHD medications have been reported to improve chronic pain as well. The patient was confirmed to have undiagnosed ADHD and was treated with the ADHD medication atomoxetine and dopamine agonist pramipexole, which dramatically improved his OD, chronic back pain, and cognitive function. Furthermore, along the course of treatment, there was improvement in cerebral blood flow in his prefrontal cortex, which was thought to reflect improved function in the region. Consequently, he was able to resume work and improve his family relationships.

**Conclusion:**

Therefore, in the cases of ODs and COPCs, screening for ADHD and, if ADHD is diagnosed, ADHD medications or dopamine agonists may be considered.

## 1. Introduction

Oral dysesthesia (OD) is a disease characterized by pain and/or abnormal sensations in the oral cavity, without any organic abnormality. Symptoms of OD include foreign body sensations, oozing, squeezing or pulling, shifting, misalignment, pain, spontaneous heat sensation, and taste (1). A special form of OD that presents with a burning sensation localized to the tongue, oral cavity, and periodontal region is known as burning mouth syndrome (BMS); OD and BMS are related disorders (1). It is also known that idiopathic oral-facial pain such as that in cases of BMS tends to coexist with chronic musculoskeletal pain, such as low back pain, even before its onset. Such coexisting idiopathic pain conditions are also called chronic overlapping pain conditions (COPCs) (2, 3). Therefore, OD is possibly a COPC. Furthermore, COPCs, such as OD, BMS, and chronic low back pain, have a similar tendency of coexisting with psychiatric disorders such as anxiety and depression, suggesting that these COPCs may have a central nervous system (CNS) dysfunction that makes the corresponding patients vulnerable to both pain and psychiatric disorders (1–5). CNS dysfunction is thought to be attributable to dysfunction of the dopaminergic nervous system (2, 4, 6). However, COPCs, such as OD, BMS, and chronic low back pain, are often refractory to treatment, and the development of novel treatment modalities tailored to their pathophysiology is expected (2, 5, 7, 8).

Recently, attention deficit hyperactivity disorder (ADHD), which is based on the dysfunction of the dopaminergic nervous system (9), has been associated with many COPCs, including headache (10), fibromyalgia (11, 12), irritable bowel syndrome (13), chronic fatigue syndrome (14), low back pain (15–17), and idiopathic orofacial pain (18–20). However, to the best of our knowledge, there are no reports of (1) ADHD comorbidity with OD or (2) of the therapeutic effects of ADHD medications or dopamine agonists on low back pain and OD. Furthermore, there are no reports evaluating cerebral blood flow over time after treatment with these medications for low back pain and OD.

We herein report the case of a patient with COPCs of OD and chronic low back pain coexistent with ADHD. A combination of ADHD medication and a dopamine agonist resulted in almost near remission of both OD and chronic low back pain and improvement in cerebral blood flow.

## 2. Case description

The patient was an 80-year-old man (height, 165 cm; weight, 65 kg). He did not have a significant family or medical history, and his personality was serious and meticulous. He was a perfectionist. His chief complaint was chronic back pain that had persisted for 25 years and an abnormal jittery sensation in his lower right gum that appeared in 2013. He was sucking his gum because of it. Abnormal gum sensation and sucking tended to worsen, which was also the case for his back pain. He lived with his wife and eldest son. He was a lawyer by profession and took a leave of absence from work in 2013 as he was unable to concentrate on his work because of his gums; his mouth would get tangled up in the gum sucking behavior. His son was in his 40s, and his unemployment bothered the patient.

**Figure 1:**
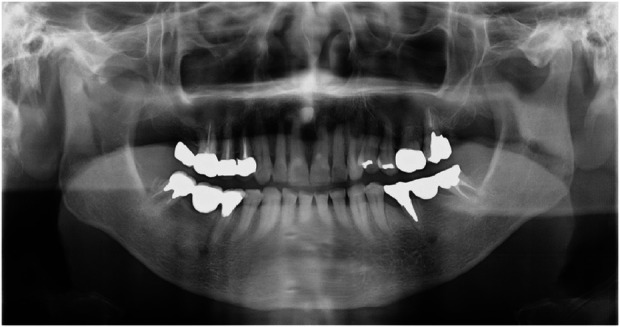
White areas with high x-ray absorption indicate cast crowns and pontics. No dental abnormalities were found that could explain the patient's persistent oral dysesthesia.

In 2012, his back pain worsened. He was often unable to get up; hence, he visited a local orthopedic surgeon. He was diagnosed with non-specific back pain with no organic abnormality. He received oral treatment with duloxetine, tramadol hydrochloride, and a fentanyl patch; however, there was no improvement. In June 2013, he visited the Department of General Medicine, Nippon Dental University Hospital, a tertiary care hospital specializing in oral-facial pain. A jaw x-ray image did not show any organic abnormality to explain the OD (Figure 1). He visited the Nippon Dental University Hospital for a year and a half and received drug therapy for OD, which included 50 mg/day of pregabalin, 0.5 mg/day of risperidone, and 2.5 mg/day of olanzapine, as well as conservative treatment, including a mouthpiece. However, he had difficulty without improvement.

Therefore, the patient was diagnosed with a suspected somatic symptom disorder with chronic back pain and OD as the main complaints. He was referred to psychiatrist SK at the Pain Clinic of the University of Tokyo Hospital in January 2015. His wife was present during the examination, and while sitting, the patient moved his hips in a fidgety manner due to his back pain and repeatedly sucked his gums throughout the examination. During examination, he complained that his back hurt so much that he could not sit up, and he would lay down on his bed to be examined. His wife stated that the stress of him not being able to tell his son to work seemed to exacerbate his OD and back pain.

**Figure 2:**
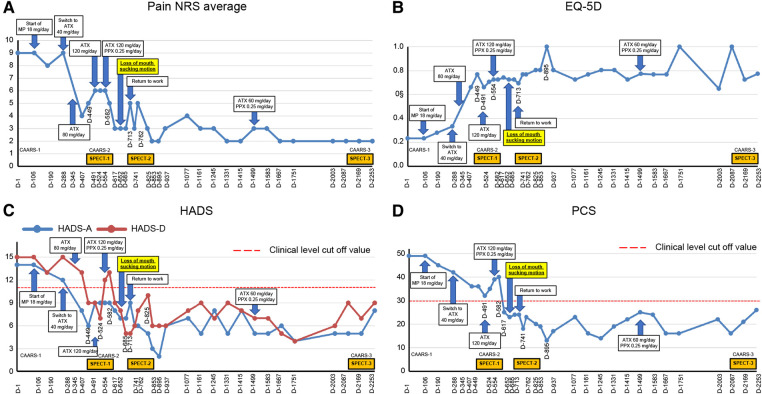
Course of treatment and objective/subjective parameters. (**A**) Pain NRS average; (**B**) EQ-5D; (**C**) HADS; and (**D**) PCS. ATX, atomoxetine; CAARS, Conners' adult ADHD rating scale; D, day; EQ-5D, euro QoL 5 Dimension; HADS-A/D, Hospital Anxiety and Depression Scale-Anxiety/Depression; MP, methylphenidate; NRS, numerical rating scale; PCS, Pain Catastrophizing Scale; PPX, pramipexole; and SPECT, single-photon emission computed tomography.

Thereafter, the patient visited our pain clinic approximately once a month, completed the questionnaire described below each time, and received feedback from Dr. SK on the results. Intensity of subjective pain was measured using the pain numerical rating scale (NRS) (21). Regarding the amount of change in the NRS scores for chronic pain, the minimum clinically important differences (MCIDs) consider a decrease of ≥2 in the NRS to be either substantial or optimal (22). Health-related aspects of quality of life (QoL) were assessed with the Euro QoL 5 Dimension (EQ-5D) (23, 24), with an EQ-5D score of 0, 1.0, and 0.08 indicating death, perfect health, and MCID, respectively (25). Symptoms of anxiety and depression were assessed using the hospital anxiety and depression scale-anxiety/depression (HADS-A/D) (26); a score of ≥8 on the HADS was considered to indicate clinical-level disease (27), and the MCID is indicated as a score of 1.5 on the HADS (28). Pain-related catastrophizing thoughts were assessed using the pain catastrophizing scale (PCS) (29); the MCID for the PCS is 6.48 points (25). The patient's average NRS score for pain in the lower back at the first visit was 9/10, with an EQ-5D score of 0.233, a HADS-A score of 14/21, a HADS-D score of 15/21, and a PCS score of 49/52 (Figure 2).

A structured interview, the mini-international neuropsychiatric interview (30), was administered to differentiate psychiatric comorbidities. The patient did not have a major depressive episode with melancholy-type features, including psychotic delusions, manic episodes, and obsessive-compulsive or psychotic disorders. Based on the diagnostic criteria of the diagnostic and statistical manual of mental disorders-5 (DSM-5) (31), he was diagnosed with somatic symptom disorder.

**Figure 3:**
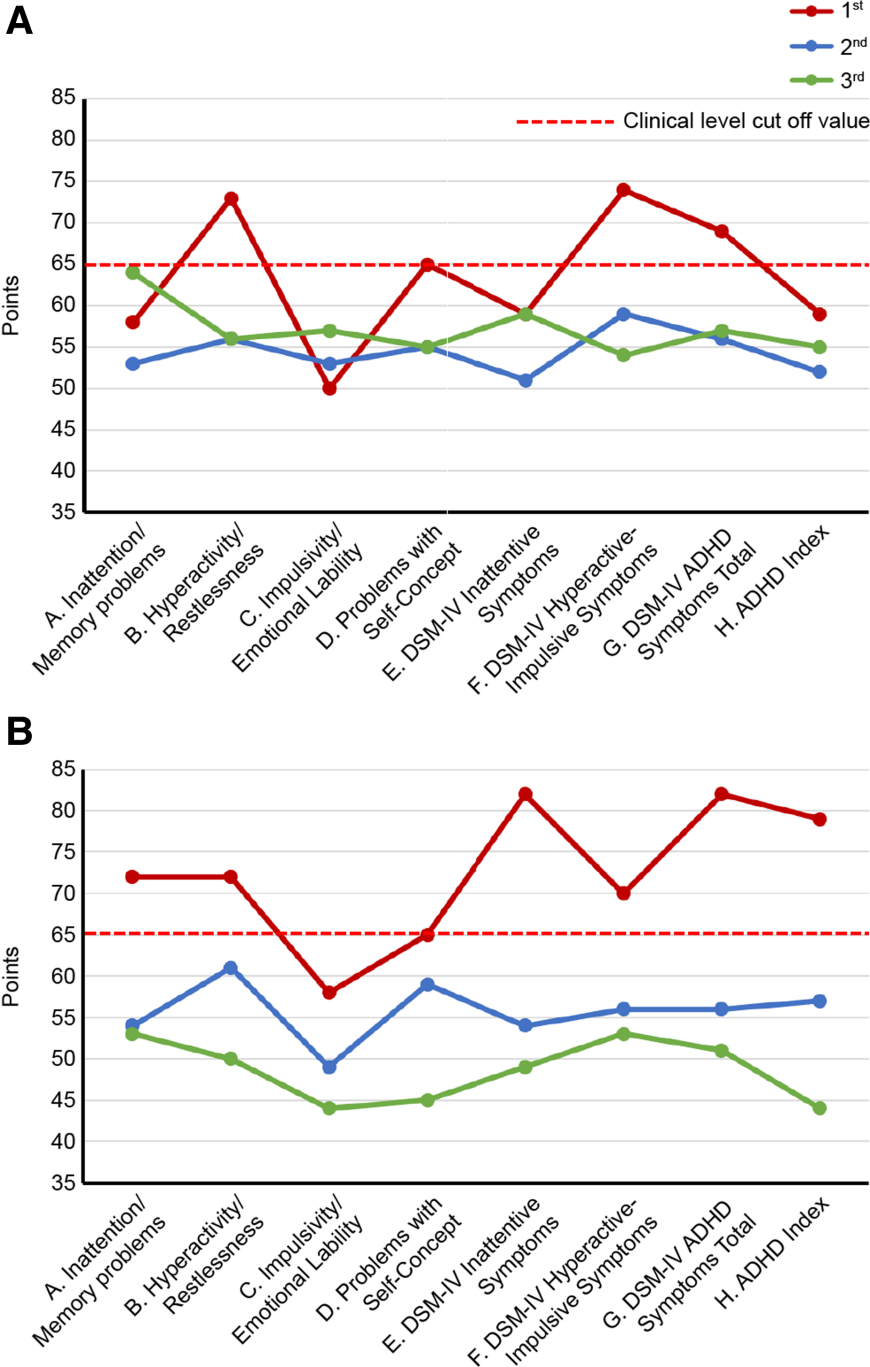
Changes in CAARS scores along the course of treatment. (**A**) CAARS-S; (**B**) CAARS-O. ADHD, Attention-Deficit/Hyperactivity Disorder; CAARS-S/O, Conners’ Adult ADHD Rating Scale Self-report/Observer rated; and DSM-IV, Diagnostic and Statistical Manual of Mental Disorders, fourth edition.

As ADHD is often comorbid with chronic pain (20), its symptom assessment and diagnosis confirmation were conducted on days 1–85. ADHD symptoms were assessed using the Conners’ adult ADHD rating scale (CAARS) self-report (CAARS-S) and observer-rated (CAARS-O) questionnaires answered by the patient and his wife, respectively (32). The patient's CAARS results showed that his subscale scores exceeded 65 points and that his ADHD symptoms were at the clinical psychiatric level (Figure 3).

ADHD was diagnosed according to the DSM-5 (31). He was easily distracted, had difficulty with organization and listening to others, and procrastinated on submitting paperwork until the very last minute before the deadline. He also had a habit of fidgeting with his hands, had an extreme dislike for sitting still or waiting in line, and tended to throw never-ending tantrums. He met the criteria for 8/9 items of inattention and 7/9 items of hyperactivity-impulsivity and was diagnosed with combined ADHD.

Day 106: After starting methylphenidate (MP) at a dose of 18 mg/day, the patient was less dazed during the day, walked without a cane, and went to museums and cafes. Day 134: When MP was increased to 36 mg/day, he began to clean up his workroom, which he previously avoided. He also started talking about his son's unemployment, a topic he had previously avoided discussing with his son, which resulted in a more intimate relationship between them. Day 288: However, he complained of insomnia and back pain after he started taking MP. The back pain intensified after the evening due to the decreased effect of MP. Hence, MP was switched to 40 mg/day of atomoxetine (ATX), another standard ADHD medication.

**Figure 4:**
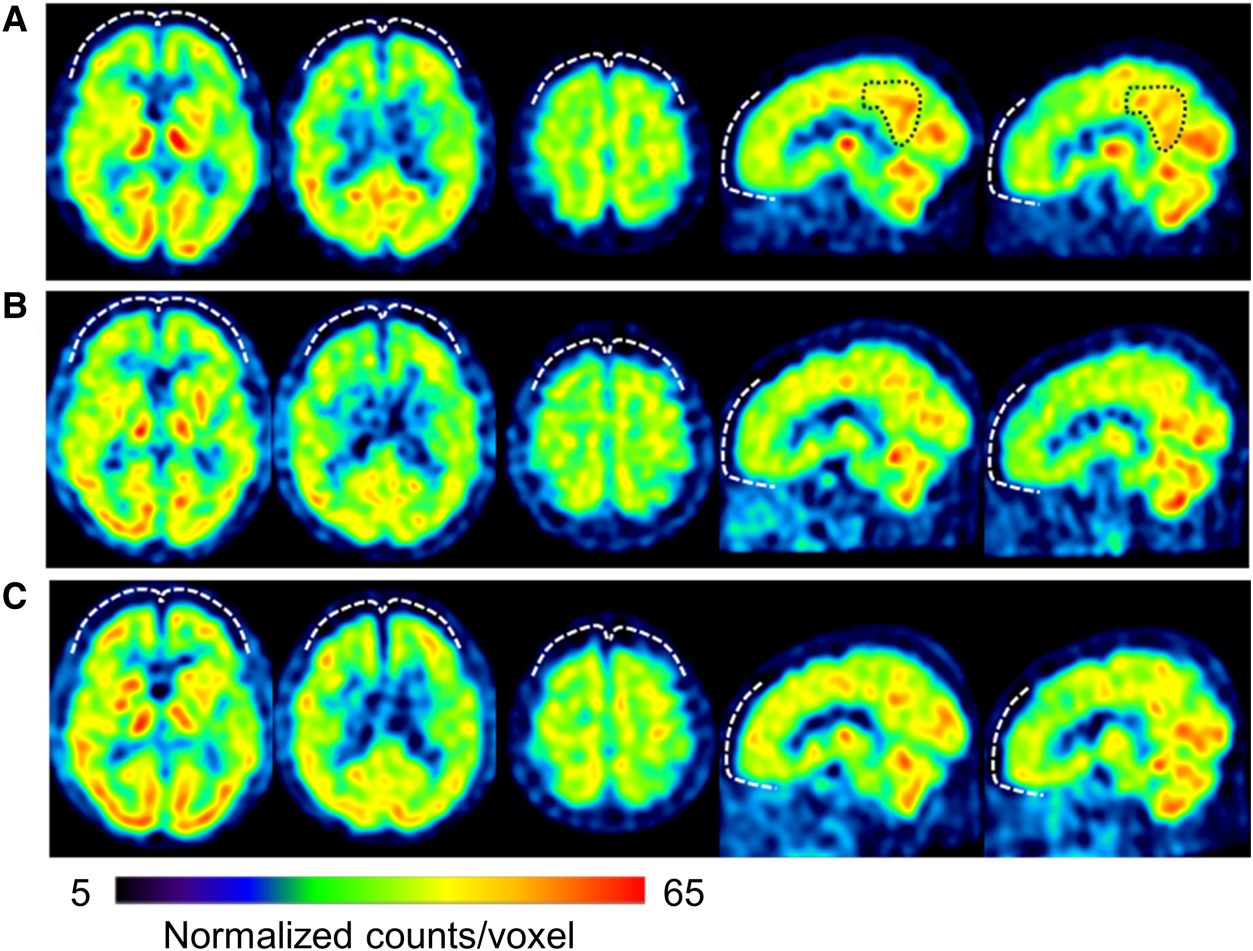
Brain perfusion SPECT images obtained on day 524 (**A**), 762 (**B**) and 2,169 (**C**), respectively. From the left side: three axial views from the basal ganglia to the frontal-parietal level, and left- and right-side sagittal views. The voxel values are normalized by the average counts-per-voxel in the cerebellum being 50, then the color bars represent from 5–60. The bilateral prefrontal cortices show hypoperfusion (white dashed curves) (**A,B**) lower than the thalamus that gradually increases to almost the same level of the thalamus and parietal-occipital lobes (**C**). The mean normalized counts in the prefrontal cortices are 40.1, 40.1 and 42.0, respectively. In contrast, the precuneus and posterior cingulate gyrus (black dashed curves) show relatively high (black dashed curves) (**A**) and decreased with time (**B,C**). Their mean normalized counts are 47.8, 43.6, and 42.2, respectively. The voxel value normalization and calculation were performed using PMOD version 3.7 (PMOD Technologies Ltd., Zurich, Switzerland). SPECT, single-photon emission computed tomography.

Day 345: The patient stated that his pain in the evening and later in the day had eased since the change to ATX. The patient's previously grim expression became brighter and softer. The dose of ATX was increased to 80 mg/day. Day 491: The patient and his wife requested to increase the dose of ATX, and the dose was increased to 120 mg/day. Day 524: The pain NRS, EQ-5D, HADS, and PCS scores showed clinically significant improvement over the MCID. All CAARS-S/O subscale scores also improved to below clinical levels (Figure 3). As the patient showed improvement in cognitive function as well as back pain, he requested a functional brain imaging study. Single-photon emission computed tomography (SPECT) was performed. 99mTc- ethyl cysteinate dimer (ECD; Fujifilm RI Pharma, Tokyo, Japan) at a dose of 740 MBq was administered to the patient resting in a spine position in a quiet room with his eyes closed. About ten minutes after injection, SPECT was performed for 30 min with a triple-head gamma camera (GCA-9300R; Canon Medical Systems Corp, Tochigi, Japan). The results showed hypoperfusion in the bilateral prefrontal cortex (Figure 4).

Day 554: The patient mentioned that he felt like the improvement with ATX had plateaued and requested to be offered a motivational medication like MP. Therefore, pramipexole (PPX), a dopamine agonist reported to be effective for ADHD and chronic pain (33, 34), was added at 0.25 mg/day in two divided doses for the morning and evening. Day 582: After starting PPX, the patient stated that he was clearly feeling better since PPX was added. He claimed that he was less sleepy during the day and was able complete tasks more quickly. Day 652: Abnormal oral sensations and gum sucking disappeared. Day 713: He resumed working as a lawyer. Day 762: A second SPECT was performed, which showed a slight improvement in cerebral blood flow compared to that in the first image (Figure 4).

Day 895: The patient started to walk 7–8 km with his son every day. Day 1,077: The patient's wife stated that the patient had an icy stare toward his family earlier. However, recently, he had become very calm. He had stopped throwing tantrums with their son and had started interacting with him calmly. Day 1,245: The patient's son had started working in the neighborhood. Day 1,331: The patient's wife reported that the patient was stubborn and serious earlier, although he has now started joking around. Day 1,415: The patient's wife reported that the patient seemed relieved that his son was able to continue working. Day 1,499: The ATX dose was reduced by half from 120 mg/day to 60 mg/day upon the patient's request. Day 2,169: CAARS and SPECT examinations were conducted for a third time; the CAARS-O scores (Figure 3) and cerebral blood flow in the prefrontal cortex showed further improvement (Figure 4). Day 2,253: The patient and his wife expressed their gratitude for the hospital's treatment, which not only improved the patient's back pain and OD, but also their familial relations. Written informed consent was obtained from the patient for participation in the study and publication of the images.

## 3. Discussion and conclusion

This case demonstrates the following three points: (1) Cases of OD may have ADHD as a comorbidity; (2) the combination of ATX (an ADHD medication) and pramipexole (a dopamine agonist) resulted in almost remission of both chronic back pain and OD; and (3) improvement in cerebral blood flow was also observed along the course of treatment.

To the best of our knowledge, this study is the first to report a case of OD concomitant with ADHD. Herein, ADHD was incidentally diagnosed. The diagnosis led to the administration of ATX and PPX, which significantly improved the patient's OD and chronic back pain of 25 years. However, adult ADHD is generally reported to be overlooked in >80% of patients, even in clinical psychiatric settings (35), and ADHD comorbid with chronic pain is even more likely to be unnoticed (20). Therefore, screening for ADHD in cases of OD is considered important.

The combination of ATX and PPX led to the near remission of both chronic low back pain and OD.

ATX is a selective noradrenaline reuptake inhibitor that acts on noradrenaline transporters in the prefrontal cortex and moderates noradrenaline and dopamine levels to improve information processing efficiency, ADHD symptoms, and pain (36, 37).

Furthermore, in this case, ATX showed improvement in low back pain, although the effect was plateaued even at the maximum dose of 120 mg/day of ATX. However, when PPX was added, back pain improved further, and OD disappeared. Dopamine in the mesolimbic system plays a central role in pain perception and down-regulatory pain inhibitory pathways. Decreased dopamine levels may enhance pain (38, 39). As a dopamine agonist, PPX has been reported to act on D2 and D3 receptors in the nucleus accumbens and enhance dopamine neurotransmission, also improving chronic pain (33). Considering that, in this case, MP, which enhances dopamine transmission in the nucleus accumbens (36), also showed improvement in low back pain, the addition of PPX may have stimulated dopamine transmission in this patient, resulting in the improvement of not only low back pain, but also OD.

The results of this case and a previous report that used aripiprazole (a dopamine partial agonist) showed improved OD (6) and suggest that dopamine agonists may be effective for OD. Furthermore, as the combination of ATX and PPX effectively treated the coexistence of low back pain and OD in the case presented herein, the improvement of both prefrontal and limbic neurotransmission may be necessary in the treatment of patients with overlapping idiopathic pain conditions, such as COPCs.

In this case, along the course of treatment, there was also improvement in cerebral blood flow in the prefrontal cortex, possibly reflecting its improved function (40). The prefrontal cortex is also functionally coupled to descending pain inhibitory pathways (41) and may serve as a filter to reduce uncomfortable stimuli, such as pain (42). The performance of the prefrontal cortex follows an inverted U-shaped curve in relation to dopamine and noradrenaline activity and is maximal when the concentration of both transmitters is moderate (42). As mentioned above, ATX enhances prefrontal cortex performance by modulating both noradrenaline and dopamine neurotransmission and serves as a virtual filter to attenuate unpleasant stimuli (42, 43). Theoretically, the improvement in blood flow in the prefrontal cortex in this case could be due to ATX. Unfortunately, SPECT was not performed in this case prior to the start of pharmacotherapy. Therefore, it is not possible to determine whether the improvement was due to ATX or PPX.

In contrast, a previous study has reported that small doses of aripiprazole improved OD and cerebral blood flow distribution imbalance; it has been stated that SPECT may be an objective therapeutic index in the treatment of OD (44). In the present case, the changes in SPECT images, which were parallel to the course of treatment, were considered to support this hypothesis.

Limitation: The improvement in OD in this case was not brought about by the addition of PPX alone. It may have been the result of the gradual effect of ATX being increased to 120 mg/day over time. Hence, one should be cautious in generalizing this treatment. Moreover, the combination treatment of ATX and PPX in this case also improved the patient's relationship with his family and social activity. Hence, the improvement in OD and cerebral blood flow may have been influenced by improvement in the psychosocial factors. Therefore the treatment effect should not be attributed solely to pharmacotherapy.

OD cases can also coexist with ADHD at a level requiring treatment, although the diagnosis is overlooked. In this case, the combination of ATX and PPX brought about near remission of both chronic low back pain and OD, as well as improvement in cerebral blood flow. ADHD comorbid with chronic pain is easily missed as a diagnosis; however, if the comorbidity diagnosis is confirmed, treatment options such as ADHD medications can be selected. Therefore, ADHD should be screened for in the practice of chronic dental pain and COPCs. This is the only case reporting an association between OD and ADHD. Further research to investigate the association between these disorders is warranted.

## Data Availability

The original contributions presented in the study are included in the article, further inquiries can be directed to the corresponding author.

## References

[1] Fortuna G Napenas J Su N Gruskha M Klasser GD. Oral dysesthesia. In: Farah C Balasubramaniam R McCullough M, editors. Oral: Contemporary book company medicine. Berlin: Springer (2019). p. 2081–105. doi: 10.1007/978-3-319-72303-7_36

[2] Maixner W Fillingim RB Williams DA Smith SB Slade GD. Overlapping chronic pain conditions: implications for diagnosis and classification. J Pain. (2016) 17(9 Suppl):T93–T107. 10.1016/j.jpain.2016.06.002 27586833 PMC6193199

[3] Herrero Babiloni A Nixdorf DR Moana-Filho EJ. Persistent dentoalveolar pain disorder: a putative intraoral chronic overlapping pain condition. Oral Dis. (2020) 26:1601–9. 10.1111/odi.13248 31797486 PMC7269821

[4] Taiminen T Kuusalo L Lehtinen L Forssell H Hagelberg N Tenovuo O Psychiatric (axis I) and personality (axis II) disorders in patients with burning mouth syndrome or atypical facial pain. Scand J Pain. (2011) 2:155–60. 10.1016/j.sjpain.2011.06.004 29913754

[5] Umezaki Y Miura A Watanabe M Takenoshita M Uezato A Toriihara A Oral cenesthopathy. Biopsychosoc Med. (2016) 10:20. 10.1186/s13030-016-0071-7 27293481 PMC4903001

[6] Karakuş İH Bulut NS. Oral cenesthopathy superimposed on burning mouth syndrome treated with aripiprazole: a case report with a phenomenological overview. Gerodontology. (2021) 38:113–6. 10.1111/ger.12516 33586237

[7] Uezato A Toyofuku A Umezaki Y Nishikawa T. Oral dysesthesia associated with autistic traits: a retrospective chart review. Eur J Oral Sci. (2019) 127:347–50. 10.1111/eos.12620 31071244

[8] Imamura Y Shinozaki T Okada-Ogawa A Noma N Shinoda M Iwata K An updated review on pathophysiology and management of burning mouth syndrome with endocrinological, psychological and neuropathic perspectives. J Oral Rehabil. (2019) 46:574–87. 10.1111/joor.12795 30892737

[9] Swanson JM Kinsbourne M Nigg J Lanphear B Stefanatos GA Volkow N Etiologic subtypes of attention-deficit/hyperactivity disorder: brain imaging, molecular genetic and environmental factors and the dopamine hypothesis. Neuropsychol Rev. (2007) 17:39–59. 10.1007/s11065-007-9019-9 17318414

[10] Salem H Vivas D Cao F Kazimi IF Teixeira AL Zeni CP. ADHD Is associated with migraine: a systematic review and meta-analysis. Eur Child Adolesc Psychiatry. (2018) 27:267–77. 10.1007/s00787-017-1045-4 28905127

[11] Yılmaz E Tamam L. Attention-deficit hyperactivity disorder and impulsivity in female patients with fibromyalgia. Neuropsychiatr Dis Treat. (2018) 14:1883–9. 10.2147/NDT.S159312 30100723 PMC6063452

[12] Kasahara S Matsudaira K Sato N Niwa SI. Pain and attention-deficit/hyperactivity disorder: the case of Margaret Mitchell. Psychosom Med. (2021) 83:492–3. 10.1097/PSY.0000000000000947 33883539 PMC8189430

[13] Hodgkins P Montejano L Sasané R Huse D. Cost of illness and comorbidities in adults diagnosed with attention-deficit/hyperactivity disorder: a retrospective analysis. Prim Care Companion CNS Disord. (2011) 13:PCC.10m01030. 10.4088/PCC.10m01030 21977356 PMC3184593

[14] Sáez-Francàs N Alegre J Calvo N Antonio Ramos-Quiroga J Ruiz E Hernández-Vara J Attention-deficit hyperactivity disorder in chronic fatigue syndrome patients. Psychiatry Res. (2012) 200(2-3):748–53. 10.1016/j.psychres.2012.04.041. 22648008

[15] Kasahara S Niwa SI Matsudaira K Sato N Oka H Fujii T High attention-deficit/hyperactivity disorder scale scores among patients with persistent chronic nonspecific low back pain. Pain Phys. (2021) 24:E299–307. 10.36076/ppj.2021/24/E299 33988951

[16] Kasahara S Matsudaira K Sato N Niwa SI. Attention-deficit/hyperactivity disorder and centralized pain: a review of the case of john F. Kennedy. Clin Case Rep. (2022) 10:e6422. 10.1002/ccr3.6422 36245472 PMC9547351

[17] Ibrahim ME Hefny MA. Central sensitization and adult attention deficit hyperactivity disorder in medical students with chronic back pain: a cross-sectional study. Egypt Rheumatol Rehabil. (2022) 49:24. 10.1186/s43166-022-00124-2

[18] Kasahara S Takahashi K Matsudaira K Sato N Fukuda KI Toyofuku A Diagnosis and treatment of intractable idiopathic orofacial pain with attention-deficit/hyperactivity disorder. Sci Rep. (2023) 13(1):1678. 10.1038/s41598-023-28931-3 36717626 PMC9887013

[19] Kasahara S Takao C Matsudaira K Sato N Tu TTH Niwa SI Case report: treatment of persistent atypical odontalgia with attention deficit hyperactivity disorder and autism spectrum disorder with risperidone and atomoxetine. Front Pain Res (Lausanne). (2022) 3:926946. 10.3389/fpain.2022.926946 35935670 PMC9353025

[20] Kasahara S Niwa SI Matsudaira K Sato N Oka H Yamada Y. Attention-deficit/hyperactivity disorder and chronic pain. Psychosom Med. (2020) 82:346–7. 10.1097/PSY.0000000000000789 32251099

[21] Jensen MP Karoly P. Self-report scales and procedures for assessing pain in adults. In: Turk DC Melzack R, editors. Handbook of pain assessment. 3rd ed. New York: Guilford Press (2011). p. 19–44.

[22] Salaffi F Stancati A Silvestri CA Ciapetti A Grassi W. Minimal clinically important changes in chronic musculoskeletal pain intensity measured on a numerical rating scale. Eur J Pain. (2004) 8:283–91. 10.1016/j.ejpain.2003.09.004 15207508

[23] Balestroni G Bertolotti G. EuroQol-5D (EQ-5D): an instrument for measuring quality of life. Monaldi Arch Chest Dis. (2012) 78:155–9. 10.4081/monaldi.2012.121 23614330

[24] Dolan P. Modeling valuations for EuroQol health states. Med Care. (1997) 35:1095–108. 10.1097/00005650-199711000-00002 9366889

[25] Suzuki H Aono S Inoue S Imajo Y Nishida N Funaba M Clinically significant changes in pain along the pain intensity numerical rating scale in patients with chronic low back pain. PLoS One. (2020) 15:e0229228. 10.1371/journal.pone.0229228 32126108 PMC7053735

[26] Zigmond AS Snaith RP. The hospital anxiety and depression scale. Acta Psychiatr Scand. (1983) 67:361–70. 10.1111/j.1600-0447.1983.tb09716.x 6880820

[27] Bjelland I Dahl AA Haug TT Neckelmann D. The validity of the hospital anxiety and depression scale. An updated literature review. J Psychosom Res. (2002) 52:69–77. 10.1016/s0022-3999(01)00296-3 11832252

[28] Puhan MA Frey M Büchi S Schünemann HJ. The minimal important difference of the hospital anxiety and depression scale in patients with chronic obstructive pulmonary disease. Health Qual Life Outcomes. (2008) 6:46. 10.1186/1477-7525-6-46 18597689 PMC2459149

[29] Sullivan MJL Bishop SR Pivik J. The pain catastrophizing scale: development and validation. Psychol Assess. (1995) 7:524–32. 10.1037/1040-3590.7.4.524

[30] Sheehan DV Lecrubier Y Sheehan KH Amorim P Janavs J Weiller E The mini-international neuropsychiatric interview (M.I.N.I.): the development and validation of a structured diagnostic psychiatric interview for DSM-IV and ICD-10. J Clin Psychiatry. (1998) 59(Suppl 20):22–33. quiz 34-57. 9881538

[31] American Psychiatric Association. Diagnostic and statistical manual of mental disorders. Washington, DC: American Psychiatric Publishing (2013). DSM-5.

[32] Conners CK Erhardt D Sparrow EP. Conners’ adult ADHD rating scale (CAARS) technical manual. North Toronto: Multi-Health Systems (1999).

[33] Holman AJ Myers RR. A randomized, double-blind, placebo-controlled trial of pramipexole, a dopamine agonist, in patients with fibromyalgia receiving concomitant medications. Arthritis Rheum. (2005) 52:2495–505. 10.1002/art.21191 16052595

[34] Kurlan R Crespi G Coffey B Mueller-Vahl K Koval S Wunderlich G A multicenter randomized placebo-controlled clinical trial of pramipexole for Tourette’s syndrome. Mov Disord. (2012) 27:775–8. 10.1002/mds.24919 22407510

[35] Ginsberg Y Quintero J Anand E Casillas M Upadhyaya HP. Underdiagnosis of attention-deficit/hyperactivity disorder in adult patients: a review of the literature. Prim Care Companion CNS Disord. (2014) 16. 10.4088/PCC.13r01600 25317367 PMC4195639

[36] Stahl SM. Stahl’s essential psychopharmacology: neuroscientific basis and practical applications. 4th ed. Cambridge: Cambridge University Press (2013).

[37] Vorobeychik Y Acquadro MA. Use of atomoxetine in a patient with fibromyalgia syndrome and attention-deficit hyperactivity disorder. J Musculoskelet Pain. (2008) 16:189–92. 10.1080/10582450802161960

[38] Wood PB. Stress and dopamine: implications for the pathophysiology of chronic widespread pain. Med Hypotheses. (2004) 62:420–4. 10.1016/j.mehy.2003.10.013 14975515

[39] Wood PB. Role of central dopamine in pain and analgesia. Expert Rev Neurother. (2008) 8:781–97. 10.1586/14737175.8.5.781 18457535

[40] Lassen NA Kanno I. The metabolic and hemodynamic events secondary to functional activation–notes from a workshop held in Akita, Japan. Magn Reson Med. (1997) 38:521–3. 10.1002/mrm.1910380402 9324315

[41] Matsuo Y Kurata J Sekiguchi M Yoshida K Nikaido T Konno SI. Attenuation of cortical activity triggering descending pain inhibition in chronic low back pain patients: a functional magnetic resonance imaging study. J Anesth. (2017) 31:523–30. 10.1007/s00540-017-2343-1 28365848

[42] Sikström S Söderlund G. Stimulus-dependent dopamine release in attention-deficit/hyperactivity disorder. Psychol Rev. (2007) 114:1047–75. 10.1037/0033-295X.114.4.1047 17907872

[43] Young JL. Chronic fatigue syndrome: 3 cases and a discussion of the natural history of attention-deficit/hyperactivity disorder. Postgrad Med. (2013) 125:162–8. 10.3810/pgm.2013.01.2631 23391682

[44] Umezaki Y Uezato A Toriihara A Nishikawa T Toyofuku A. Two cases of oral somatic delusions ameliorated with brain perfusion asymmetry: a case report. Clin Neuropharmacol. (2017) 40:97–9. 10.1097/WNF.0000000000000207 28225385 PMC5349303

